# Secondary Metabolites with Anti-Inflammatory Activities from an *Actinobacteria* *Herbidospora daliensis*

**DOI:** 10.3390/molecules27061887

**Published:** 2022-03-14

**Authors:** Jih-Jung Chen, Tzong-Huei Lee, Ming-Jen Cheng

**Affiliations:** 1 Department of Pharmacy, School of Pharmaceutical Sciences, National Yang Ming Chiao Tung University (NYCU), Taipei 11221, Taiwan; jjungchen@nycu.edu.tw; 2 Department of Medical Research, China Medical University Hospital, China Medical University, Taichung 404, Taiwan; 3 Institute of Fisheries Science, National Taiwan University, Taipei 10617, Taiwan; thlee1@ntu.edu.tw; 4 Bioresource Collection and Research Center (BCRC), Food Industry Research and Development Institute (FIRDI), Hsinchu 30062, Taiwan

**Keywords:** *Herbidospora* *daliensis*, streptosporangiaceae, triterpenoids, anti-inflammatory activities

## Abstract

Bioassay-guided fractionation of extracts derived from solid cultures of a *Herbidospora* *daliensis* originating from Taiwan led to the isolation of five new compounds, for which we propose the name herbidosporadalins A–E (**1**–**5**), one isolated for the first time, herbidosporadalin F (**6**), together with two known compounds (**7** & **8**). Their structures were elucidated by spectroscopic analyses, including 1D- and 2D-NMR experiments with those of known analogues, and on the basis of HR-EI-MS mass spectrometry, their anti-inflammatory activities were also evaluated. Of these isolates, herbidosporadalin A (**1**), B (**2**), F (**6**) and G (**8**) showed NO inhibitory activity, with IC_50_ values of 11.8 ± 0.9, 7.1 ± 2.9, 17.8 ± 1.7, and 13.3 ± 6.5 μM, stronger than the positive control quercetin (IC_50_ = 36.8 ± 1.3 μM). To the best of our knowledge, this is the first report on 3,4-seco-friedelane metabolites (**5**, **6** & **8**) from the genus *Herbidospora*.

## 1. Introduction

*Actinobacteria* are a very special group of microorganisms that mainly grow in soil and can also be found in oceans, streams, lake water or sediment, animals, plants, nodules, compost, hot springs, geothermal and other environments. Its morphology is as varied as that of fungi, and it has structures such as substrate hyphae, aerial hyphae, spore, spore chain and sporangium in its life history. However, it resembles prokaryotes-like bacteria in physiological structure, so it has been mistaken for fungi for a long time, and some people regard it as a microorganism between bacteria and fungi-boundary microorganisms. *Actinobacteria* themselves have the ability to produce a variety of physiologically active products [1,2,3,4,5,6,7]. Therefore, it plays a key role in the pharmaceutical industry and the food industry. Our team has also isolated and collected *actinobacteria* resources from all over Taiwan and various environments over the years. In addition to the common *Streptomyces*, we have also discovered many new species from different environments throughout Taiwan. The goal is to isolate new compounds from new species, with the hope of discovering special compounds from these new species.

Our team has collected more than 1500 Taiwan native *actinobacteria* in the past 20 years. They have collected soil samples from various environments in Taiwan, including paddy fields, vegetable gardens, orchards, weeds, hot springs, culture pond sediments, lake sediments, mangroves, and wetland. After appropriate pretreatment, the *actinobacteria* were isolated by serial dilution and selective medium (HV agar). In the past, our team published 5 new native species in IJSEM [8,9,10,11], one of which was *Herbidospora daliensis*, which was isolated from the sediments of lakes in central Taiwan [11]. According to the database literature search, the past research of *Herbidospora* genus focused on the classification of molecular biology [12,13,14]. There are few studies on natural chemistry [15]. Recently, through the anti-inflammatory activity-screening platform, it was found that the *Herbidospora* daliensis *strain* is an actinomycete with strong anti-inflammatory activity. Our research is unique and original compared with the literature [1,2,3,4,5,6,7], and the components secreted by the bacteria are worthy of in-depth research and discussion.

**Figure 1:**
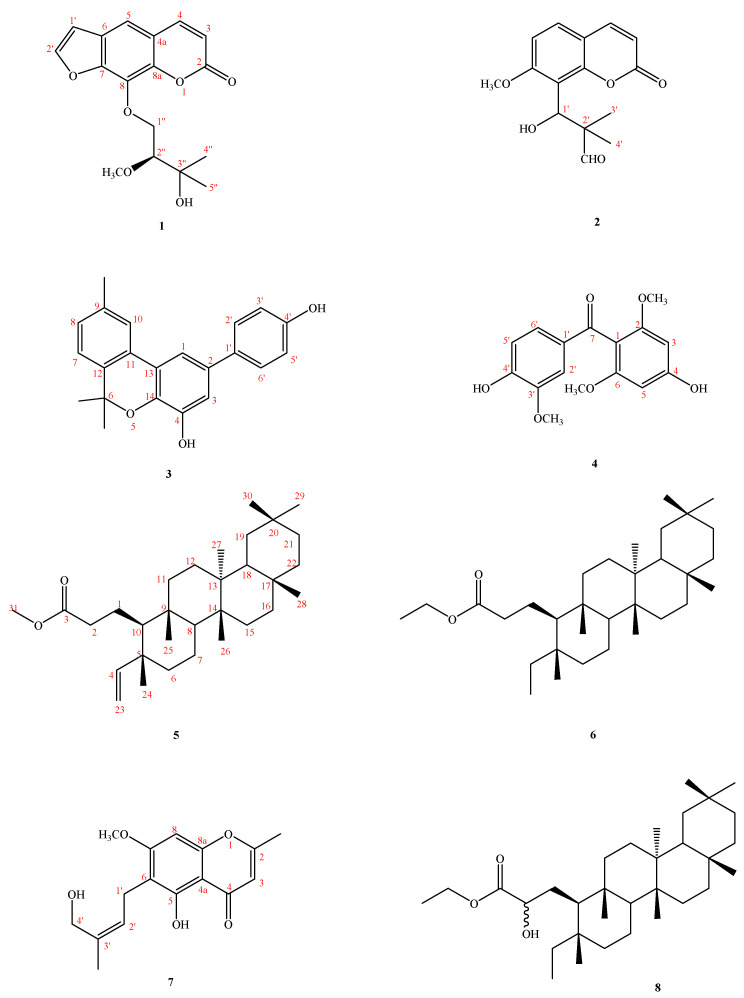
Compounds **1–8**, isolated from *Herbidospora daliensis*.

**Figure 2:**
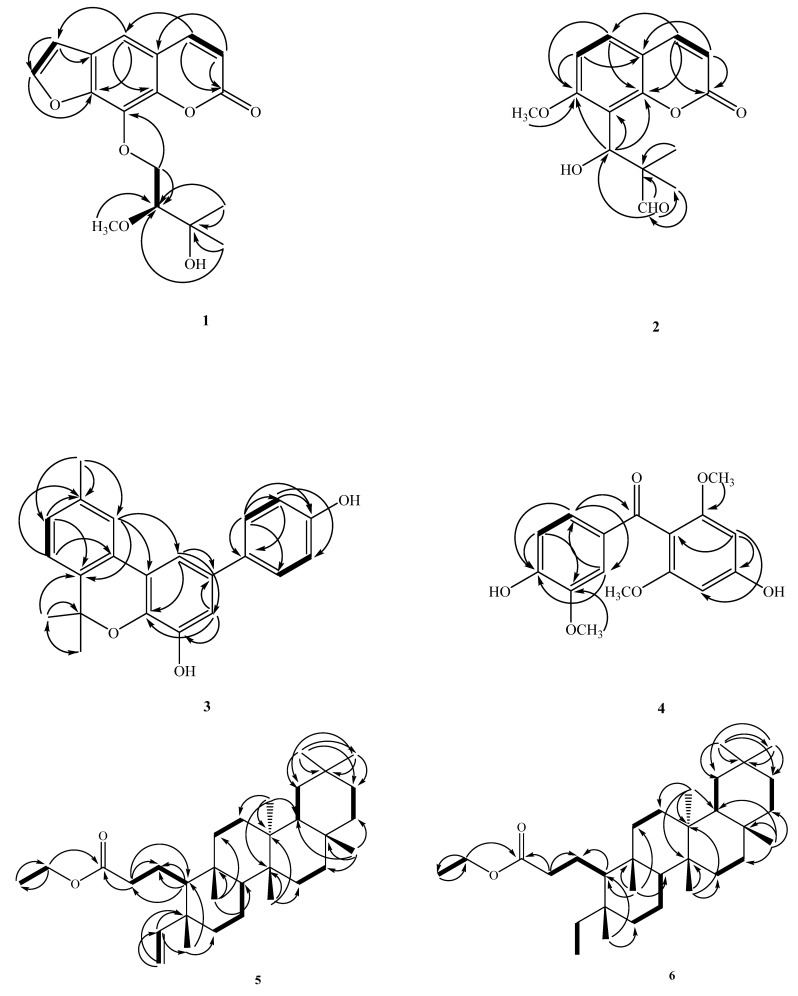

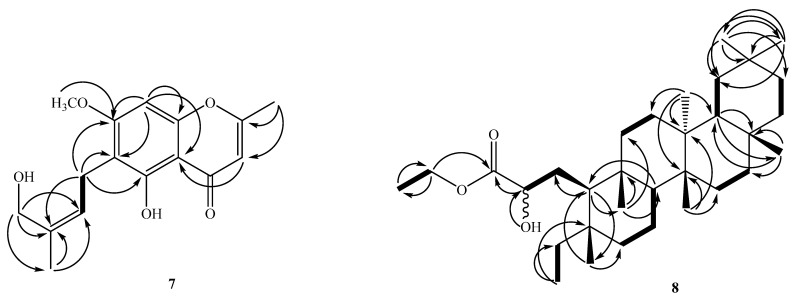
Key COSY (▬) and HMBC (→) correlations of **1**–**8**.

**Figure 3:**
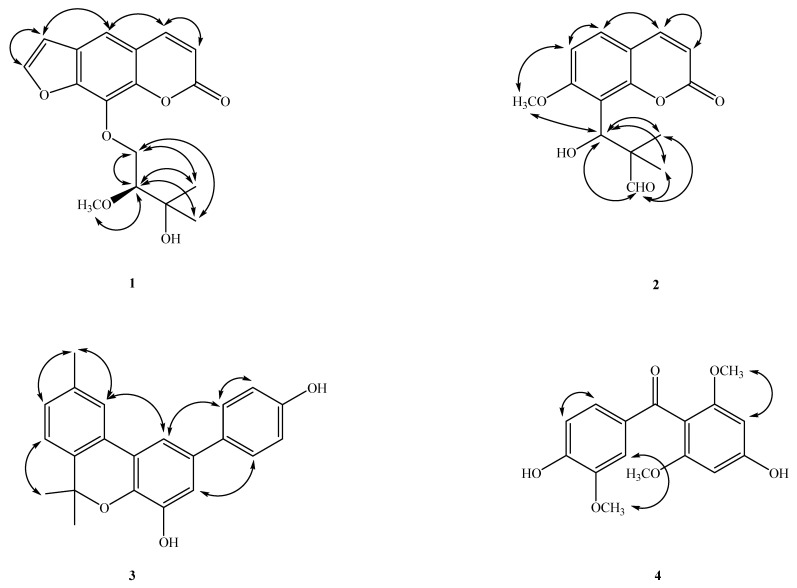

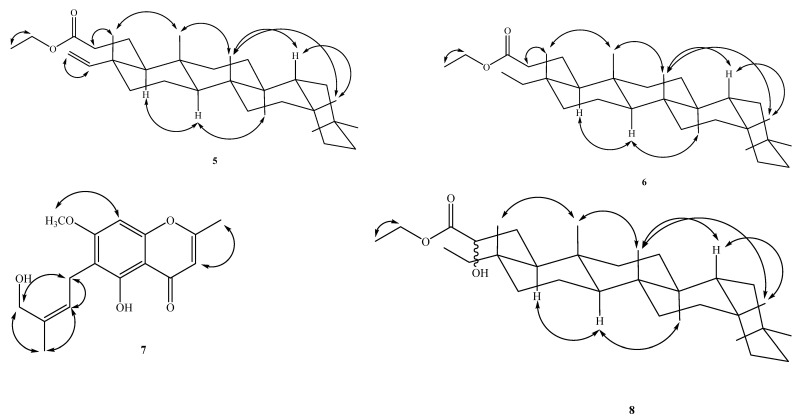
Major NOESY (↔) contacts of **1**–**8**.

Careful examination of the constituents and the anti-inflammatory principles of this material has led to the isolation and characterization of five other new ones, two of which contain coumarin moiety: herbidosporadalin A (**1**) and herbidosporadalin B (**2**), and herbidosporadalin C (**3**), herbidosporadalin D (**4**), and herbidosporadalin E (**5**), as well as three known compounds (Figure 1). The structures of these compounds were determined through spectral analyses (Figure 2 and Figure 3). The structural elucidation of **1**–**6** and the anti-inflammatory of the isolates are described herein.

## 2. Results and Discussion

### 2.1. Structural Elucidation of Compounds

Compound **1**, obtained as amber oil with [α]^25^_D_: +118.82 (*c* 0.04, CHCl_3_)., had the molecular formula C_1__7_H_1__8_O_6_, as determined by HR-EI-MS data (*m*/*z* 318.1105 ([M + H]^+^; calc. 318.1103)) in combination with its ^1^H-NMR, ^13^C-NMR and DEPT, requiring nine degrees of unsaturation (DBE). The UV absorption bands at λmax 337 (3.81), 298 (4.24), 263 (4.29), 248 (4.48) nm were characteristic of the coumarin skeleton and the IR spectrum revealed the presence of a hydroxyl group (3482 cm^−1^) and one carbonyl group (1725 cm^−1^). The ^1^H and ^13^C NMR data (Table 1) of **1** showed signals similar to those of heraclenol [16], except for an additional methoxy group [δ_C_/δ_H_ 49.3/3.24(3H, s, OCH_3_-2″)]. The ^1^H NMR spectrum indicated that **1** possessed a coumarin nucleus based on a pair of characteristic doublets at δ_H_ 7.75 (1H, *d*, *J* = 9.6 Hz, H-4) and 6.35 (1H, d, *J* = 9.6 Hz, H-3), one singlet aromatic proton at δ_H_ 7.36 (1H, s, H-5), two doublets benzofuran protons at δ_H_ 6.80 (1H, d, *J* = 2.0 Hz, H-3H) and 7.68 (1H, d, *J* = 2.0 Hz, H-2H), two Me groups at δ_H_ 1.26 (s, CH_3_-4 CH(grou, an oxymethylene protons at δ_H_ 4.37 (1H, dd, *J* = 10.0, 8.4 Hz, H-1H’) and 4.71 (1H, dd, *J* = 10.0, 2.8 Hz, H-1H’), two Me groups at δ_H_ 1.26 (s, CH_3_-426 (s, CH, one oxymethine at δ_H_ 4.00 (1H, dd, *J* = 8.4, 2.8 Hz, H-2″) and one aliphatic methoxy group at δ_H_ 3.24 (3H, s, OCH_3_-2″). The ^13^C NMR and DEPT spectra of **1** displayed 17 carbons, including one lactone carbonyl carbon at δ_C_ 210.6; two olefinic methines at δ_C_ 106.8 (C-3′) and 146.8 (C-2′); 8 aromatic carbons at δ_C_ 148.2 (C-7), 144.3 (C-4), 143.5 (C-8a), 131.9 (C-8), 126.0 (C-6), 116.5 (C-4a), 114.7 (C-3), and 113.5 (C-5); two methyls at δ_C_ 21.4 (C4″) & 20.6 (C-5″); one methoxy at δ_C_ 49.3 (OCH_3_-2″); an adjacent oxymethylene at δ_C_ 75.6 (C-1″); and an oxygenated quaternary carbon at δ_C_ 75.5 (C-3″). In the HMBC spectrum, the conjugated lactone carbonyl carbon (δ_C_ 160.0, C-2) revealed correlations with H-3 (δ_H_ 6.35) and H-4 (δ_H_ 7.75), one oxygenated quaternary carbon C-8a (δ_C_ 143.5) showed correlations with H-4 (δ_H_ 7.75) & H-5 (δ_H_ 7.36), and the other oxygenated quaternary carbon (δ_C_ 148.2, C-7) showed the correlations with H-2′ (δ_H_ 7.68), H-5 (δ_H_ 7.36) and H-3′ (δ_H_ 6.80). These 1D and 2D NMR spectroscopic data suggested the existence of an alkyl butyl substituent at C-8 and a methoxy group at C-2″. The deshielded methylene signal appeared at δ_H_ 4.37 (1H, dd, *J* = 10.0, 8.4 Hz, H-1″) and 4.71 (1H, dd, *J* = 10.0, 2.8 Hz, H-1″), which was correlated to carbon (δ_C_ 75.6, C-1″) from the HSQC spectrum. The tertiary carbon (δ_C_ 76.0, C-2″) revealed interactions with (CH_3_)_2_-4″ and 5″ (δ_H_ 1.26), CH_2_-1″ (δ_H_ 4.37/4.71), and OCH_3_-2″ (δ_H_ 3.24), and an oxygenated quaternary carbon (δ_C_ 75.5, C-3″) revealed interactions with the Me group (δ_H_ 1.26, CH_3_-4″ & 5″) and oxymethine (δ_H_ 4.00, H-2″) in the HMBC spectrum. These data and HMBC correlations showed unambiguously the structure of the alkylbuty unit as a 3-hydroxy-2-methoxy-3-methylbutoxy group. Furthermore, compound **1** showed dextrorotatory optical activity with [α]^2^^5^_D_ + 111.8 (*c* 0.04, CHCl_3_), and the absolute configuration of C-2″ was proposed as *S*-form after comparison with the (*R*)-heraclenol [16]. On the basis of the above evidence, the structure of **1** was (S)-9-(3-hydroxy-2-methoxy-3-methylbutoxy)-7*H*-furo [3,2-g]chromen-7-one and was named herbidosporadalin A.

Compound **2** was obtained as an optically inactive yellow oil. [α]^2^^5^_D_ ± 0 (*c* 0.32, CHCl_3_). The molecular formula was established as C_1__5_H_16_O_5_ by EIMS and HRESIMS analysis (276 [M]^+^). The UV absorption band at λ_max_ 321 (4.45), 258 (3.94), 248 (3.91), and 222 (4.30) nm were characteristic of the coumarin skeleton and the IR (1732 cm^−1^), and aromatic moiety (1606, 1566 cm^−1^). The ^1^H NMR spectrum (Table 1) was similar to that of murrayacarpin A [17], but a 1-hydroxy-2,2-dimethyl-3-oxopropyl group [δ_H_ 1.06/1.07 (each 3H, s, CH_3_-3′ & 4′), 5.52 (1H, s, H-1′), 9.71 (1H s, CHO-2′)] in **2** was substituted for an ethyl group [δ_H_ 0.88 (3H, t, *J* = 7.2 Hz, H-17), 1.26 (br s, H-16)] in murrayacarpin A. ^13^C NMR spectrum (Table 2) also supported the presence of a 1-hydroxy-2,2-dimethyl-3-oxopropyl group [δ_C_ 18.4 (C-3′), 20.0 (C-4′), 52.8 (C-2′), 72.0 (C-1′), 203.8 (CHO-2′)].

**Scheme 1:**
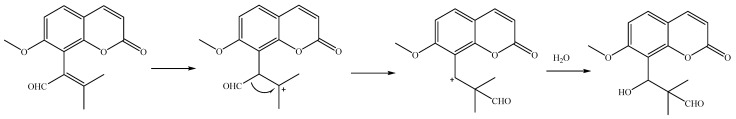
The possible biosynthetic pathway of **2**.

The associations of the HMBC signals are as follows: H-1′ (δ_H_ 5.52) is associated with C-7, 8, 9; δ_H_ 9.71 (s) is associated with C-2′, 3′, 4′; and it was determined that the branched chain of compound **2** contains a *tert*-butyl group, and the mass peak at *m*/*z* 205 (100) seen in the EI-MS spectrum was presumed to be the fragment of the molecular parent peak removed -CCHO(CH_3_)2-. Compound **2** is as follows: 7-methoxy-8-(1-hydroxy-2,2-dimethyl-3-oxopropyl)coumarin, designated as herbidosporadalin B. Tertiary butyl is very rare in nature, and it has been found in compounds isolated from *Ginkgo biloba* in the past. The structure of **1** is a quite interesting new skeleton, and its possible biosynthesis pathway is speculated, as shown in Scheme 1.

Compound **3** was obtained as oil, and the high-resolution electron impact mass spectrometry (HR-EI-MS) data determined the molecular formula to be C_22_H_20_O_3_ (*m*/*z* 332.1429 ([M]+, calcd 332.1412)), indicating 13 degrees of unsaturation. The ^13^C NMR (Table 2) and DEPT spectra exhibited 22 signals for three methyl, nine methines and ten quaternary carbons. UV absorption of benzenoid nucleus at 287 (4.39), 265 (4.60), and 212 (4.59) nm and the bands of hydroxyls (3390 cm^−1^), and aromatic (3023, 1598 and 1504 cm^−1^) functionalities in the IR spectrum together with the ^1^H-NMR spectrum of **3** showed characteristics of a chromene ring, i.e., of the ABX-pattern H-atoms H-7 (δ_H_ 7.13 (1H, d, *J* = 7.9 Hz), H-8 (δ_H_ 7.12 (1H, br d, *J* = 7.9 Hz), H-10 (δ_H_ 7.57 (1H, br s)), CH_3_-9 (δ_H_ 2.38 (3H, s), of a pair of magnetically equivalent Me groups (δ_H_ 1.65 (s, Me-6, 6)). The base peak at *m*/*z* 317 ([M-Me]^+^) in the EI-MS and a set of signals at δ_C_ 27.7 (Me(6 × 2)), 77.3 (C(3′), 128.2 (C-11), 136.4 (C-12), 123.2 (C-7), 128.9 (C-8), 137.4 (C-9), and 123.1 (C-10) in the ^13^C-NMR spectrum provided further support for the presence of a geminally dimethyl-substituted chromene system. The remaining unsaturation degrees suggested the presence of a typical biphenyl unit that was supported by the remaining 12 aromatic C-atom signals in the ^13^C-NMR spectrum. The existence of two phenolic OH groups was confirmed by the signals of two exchangeable H-atoms in the ^1^H-NMR (δ_H_ 5.42 and 4.87) and two oxygenated aromatic C-atoms in the ^13^C-NMR spectrum (δ_C_ 144.8 (C-3) and 154.8 (C-10). The ^1^H-NMR spectrum showed a set of ds of *ortho*-coupled H-atoms at δ_H_ 7.48 (d, *J* = 8.6 Hz, H-2′, 6′) and 6.88 (d, *J* = 8.6 Hz, H-3′, 5′)), typical for a *para*-substituted aryl moiety. Therefore, one OH group was assigned to C-10. A set of doublets of *meta*-coupled H-atoms at δ_H_ 7.06 (d, *J* = 2.0 Hz, H-3) and 7.40 (d, *J* = 2.0 Hz, H-1) belonged to a 1,2,3,5-tetrasubstituted benzene moiety (ring A). A HMBC experiment allowed positioning of the substituents at ring A. The H-10 signal at δ_H_ 7.57 showed a two-bond connectivity with C-11 (δ_C_ 128.2), and a three-bond connectivity with C-12 (δ_C_ 136.4), C-13 (δ_C_ 122.6) and C-11 (δ_C_ 128.2) in the HMBC plot (Figure 2). H-3 at δ_H_ 7.00 showed a two-bond connectivity with C-4 (δ_C_ 145.8), and a three-bond connectivity with C-14 (δ_C_ 138.5), C-1 (δ_C_ 112.4), and C-1′ (δ_C_ 134.5), which suggested that the second OH group was attached to C-3. Further support for the determination of the structure was provided by the signal of H-1 at δ_H_ 7.40, showing a two-bond connectivity with C-13 (δ_C_ 122.6), and a three-bond connectivity with C-3 (δ_C_ 112.9), C-14 (δ_C_ 138.5), and C-1′ (δ_C_ 134.5) in the HMBC plot. The ^1^H- and ^13^C-NMR (Table 2) and HMBC data (Figure 2) established the structure of herbidosporadalin C (**3**) as 2-(4-hydroxyphenyl)-6,6,9-trimethyl-6*H*-benzo[*c*]chromen-4-ol.

Compound **4** was isolated as an amorphous powder, and its molecular formula was established as C_1__6_H_16_O_6_ by HREIMS. The UV spectrum exhibited bands at 233 sh, 286 nm, suggesting the presence of a benzophenone moiety. A band attributable to ketone (1729 cm^−1^) was observed in the IR spectrum. The ^1^H NMR data (Table 3) of **4** showed an ABX system in a benzene ring [δ_H_ 7.59 (d, *J* = 1.6 Hz, H-2′) 7.22 (dd, *J* = 8.3,1.6 Hz, H-6′), 6.84 (d, *J* = 8.3 Hz, H-5′)], two symmetrical aromatic protons [δ_H_ 6.08 (2H, s, H-3 & 5′)] in another benzene ring, the existence of two phenolic OH groups was confirmed by the signals of two exchangeable H-atoms in the ^1^H-NMR (δ_H_ 6.02 and 5.32) and two oxygenated aromatic C-atoms in the ^13^C-NMR spectrum (δ_C_ 150.5 (C-4′)) and 158.5 (C-4), and three methoxy groups [δ_H_ 3.93 (3H, s, OCH_3_-3′), 3.63 (6H, s, OCH_3_-2, 6)], indicating the presence of a 4-hydroxy-3-methoxyphenyl moiety and a 4-hydroxy-2,6-dimethoxyphenyl.

These two phenyl moieties were connected by a C=O group [δ_C_ 194.2 (C-7)]. The HMBC spectrum (Figure 2) revealed correlations between H-2′ (δ_H_ 7.59), H-6′ (δ_H_ 7.22)/C-7 (δ_C_ 194.2), OCH_3_-3′ (δ_H_ 3.93)/C-3′(δ_C_ 146.5), OCH_3_-2 & 6 (δ_H_ 3.63)/C-2 & 6 (δ_C_ 158.6), H-5′ (δ_H_ 6.84)/C-1′ (δ_C_ 131.1), C-3′ (δ_C_ 146.5), C-4′ (δ_C_ 150.5), H-6′ (δ_H_ 7.22)/C-2′ (δ_C_ 110.1), C-4′ (δ_C_ 150.5), C-7 (δ_C_ 194.2), H-2′ (δ_H_ 7.59)/C-4′ (δ_C_ 150.5), C-6′ (δ_C_ 126.2), C-7 (δ_C_ 194.2), H-3 (δ_H_ 6.08)/C-1 (δ_C_ 110.4), C-2 (δ_C_ 158.6), C-4 (δ_C_ 158.5), and C-5 (δ_C_ 92.0). This result was further supported by the key NOESY data (Figure 3), i.e., by the correlations δ_H_ 7.59 (H-2′)/OCH_3_-3′ (δ_H_ 3.93), δ_H_ 7.22 (H-6′)/6.84 (H-5′), and δ_H_ 6.08 (H-3)/OCH_3_-2 (δ_H_ 3.63). Thus, the biphenylpropanoid structure of **4** was elucidated as 4,4-dihydroxy-2,6,3′-trimethoxy-benzophenone, which was confirmed by DEPT, COSY, HSQC, NOESY (Figure 2) and HMBC (Figure 1) experiments, and was named herbidosporadalin D.

The structure elucidation of three secotriterpenes compounds **6**–**8** was based on the assignments of the NMR spectra, which was confirmed by the 2D experiments (COSY, HSQC, HMBC and NOESY). The ^1^H and ^13^C chemical shifts of the hydrogen and carbon atoms are given in Table 1 and Table 2.

Compound **5** was obtained as a light white solid with a 197–198 °C melting point, and the high resolution electron impact mass spectrometry (HR-EI-MS) data determined the molecular formula to be C_32_H_54_O_2_ (*m*/*z* 470.4117 ([M]^+^, calcd 470.4124)), indicating six degrees of unsaturation. The IR spectrum indicated the presence of carbonyl (1736 cm^−1^), and double bond (1633 cm^−1^) functionalities. The ^1^H NMR spectrum of **5** (CDCl_3_, 500 MHz) showed seven methyl singlets resonating at δ_H_ 1.15 (3H, s, CH_3_-28), 0.99 (3H, s, CH_3_-27), 0.97 (3H, s, CH_3_-30), 0.97 (3H, s, CH_3_-26), 0.96 (3H, s, CH_3_-24), 0.92 (3H, s, CH_3_-29), 0.86 (3H, s, CH_3_-25), and one triplet at 1.23 (3H, t, *J* = 7.2 Hz, CH_3_-32), respectively, on the basis of comparison with those reported in the literature for closely related triterpenes.

The ^1^H-NMR and ^13^C-NMR spectra (Table 2) of **5** were similar to those of 3,4-seco-friendelan-3-oic acid, except that an ethoxy group [δ_H_ 4.08 (q, *J* = 7.2 Hz, H-31) and 1.23 (t, *J* = 7.2 Hz, H-32)] and the terminal double bond [δ_H_ 5.60 (dd, *J* = 17.4, 10.8 Hz, H-4), 4.90 (dd, *J* = 10.8,1.1 Hz, H-23a), 4.88 (dd, *J* = 17.4,1.1 Hz, H-23)] of **5** replaced a hydroxyl group at C-3 and a single bond at C-4–C-23 [δ_H_ 1.14/1.37 (2H, m, H-4), 0.78 (t, *J* = 7.5 Hz, H-23)] of 3,4-seco-friendelan-3-oic acid.

The signal of C-5 showed a correlation with hydrogens CH_3_-24 (δ_H_ 0.96) and these hydrogens with C-4 (δ_C_ 42.0), C-10 (δ_C_ 58.3) and C-6 (δ_C_ 41.5); C-10 (δ_C_ 58.3) with CH_3_-25 (δ_H_ 0.86); CH_3_-25 with C-8 (δ_C_ 53.0), C-9 (δ_C_ 38.6) and C-11 (δ_C_ 35.1); C-8 (δ_C_ 53.0) with CH_3_-26 (δ_H_ 0.97); CH_3_-26 (δ_H_ 0.97) with C-13 (δ_C_ 39.6), C-14 (δ_C_ 38.3) and C-15 (δ_C_ 32.2); C-13 and C-14 with CH_3-_27 (δ_H_ 0.99); CH_3-_27 (δ_H_ 0.99) and CH_3_-30 (δ_H_ 0.97) with C-19 (δ_C_ 35.2), C-20 (δ_C_ 28.1), and C-21 (δ_C_ 32.7). The relative configuration of 1 was determined on the basis of a NOESY experiment (Figure 3). Therefore, the structure of **5** was determined as shown and given the name as herbidosporadalin E.

The molecular formula of compound **6**, C_32_H_56_O_2_, was established by HREIMS [*m*/*z* 472.4276, (M + H)^+^]. Its IR spectrum showed carbonyl absorption at 1737 cm^−1^. The ^1^H and ^13^C NMR data (Table 1 and Table 2) for **6** were similar to those of 2-hydroxyl-3,4-seco-friedelan-3-oic acid ethyl-ester (**8**). The main differences between them were that the oxymethine group [δ_H_ 4.07 (C-2)] at C-2 in **8** was replaced by a methylene group after observing HMBC correlations from CH_2_-2 (δ_H_ 2.29 (t, *J* = 8.7 Hz) to C-3 (δ_C_ 173.8) and C-1 (δ_C_ 21.0). Compound **6** was first isolated from a natural source, though it has since been synthesized [18]. Therefore, the structure of **6** was established as herbidosporadalin F, as shown.

Compound **7** was obtained as colorless oil. The molecular formula was determined to be C_16_H_18_O_5_ from the quasi-molecular ion peak [M + H]^+^ at *m*/*z* 290.1144 by HR-EI-MS (calcd. for C_16_H_18_O_5_Na, 290.1154), corresponding to 8 degrees of unsaturation. The IR spectrum exhibited absorptions of a strong intermolecular hydrogen bonding at 3416 cm^−1^ and a conjugated carbonyl group (1660 cm^−1^). The ^1^H and ^13^C NMR data (Table 1) displayed the presence of mono-substituted benzene signals [δ_H_ 6.38 (1H, s, H-8)/δ_C_ 162.1 (C-7), 157.4 (C-5), 156.0 (C-8a), 111.4 (C-6), 105.0 (C-4a), and 89.7 (C-8)], an olefinic methine [δ_C_/δ_H_ 108.6/6.03 (1H, s, H-3)], an olefinic methyl [δ_C_/δ_H_ 20.9/2.34 (3H, s, CH_3_-2)], a carbonyl carbon signal (δ_C_ 181.7, C-4), and a carbon signal (δ_C_ 165.9, C-2). Furthermore, the characteristic UV absorption signals at 291, 256, and 232 nm for the chromone skeleton and the above-mentioned NMR data indicated that compound **7** showed signals and coupling patterns similar to those of cnidimol A [19]. The molecular formula C_16_H_18_O_5_ of **3** displayed more 14 units than cnidimol A, and NMR data was presented a methoxy group [δ_C_/δ_H_ 56.1/3.89 (3H, s, OCH_3_-7). In the HMBC spectrum, the deshielded methylene protons H-1′ (δ_H_ 3.40) showed the correlations to the quaternary carbons at C-3′ (δC 134.8), C-6 (δ 111.4), C-5 (δ_C_ 157.4), and C-7 (δ_C_ 162.1), whereas the olefic proton at H-2′ (δ_H_ 5.32) was correlated to methylene carbon at C-1′ (δ_C_ 21.4) and oxymethylene at C-4′ (δ_C_ 61.7), and the methyl proton at H-5′ (δ_H_ 1.77) was correlated with C-2′ (δ_C_ 124.7), C-3′ (δ_C_ 134.8), and C-4′ (δ_C_ 61.7). Thus, the data indicated a hydroxyprenyl at C-6. Moreover, the HMBC spectrum featured cross peaks long-range correlations from H_3_-7 (δ_H_ 3.89) to C-7 and from CH_2_-4′ (δ_H_ 4.25) to C-3′ (δ_C_ 134.8), suggesting the methoxy group at C-7 and a hydroxyethyl group at C-3′. In the NOESY spectrum of **7**, there was a correlation between the protons of 4′. In the NOESY spectrum of **7**, a correlation between the protons of H-2′/H-5′ and H-1′/H-4′ provided evidence for the (2′*Z*)-geometry (Figure 1), which was also confirmed by the chemical shift for 5′-CH3 (δ_C_ 22.5) [20,21]. Therefore, compound **7** was 5-hydroxy-6-[(2′*Z*)-4′-acetoxy-3′-methylbut-2′-enyl]-7-methoxy-2-methylchromone [22].

Compound **8**, m.p. 211–212 °C, was obtained as a white, amorphous solid. The molecular formula was deduced to be C_32_H_56_O_3_ from its NMR spectral data. Ester carbonyl and hydroxyl groups were indicated by absorption bands at 1752 and 3400 cm^−1^ in the IR spectrum, respectively. The ^13^C NMR spectrum of 7 (in CDCl_3_) indicated the presence of 32 signals, seven corresponding to quaternary, four to methine, 12 to methylene and nine to methyl carbons on the basis of the DEPT experiment. The spectral features indicated that 7 has a similar molecular framework to **5**. The absence of a signal corresponding to a methylene group around δ_C_ 37.4/δ_H_ 2.25 (C-2), the presence of a CHOH group at δ_H_ 4.07/δ_C_ 71.8 (C-2), and a terminal ethyl group [δ_H_ 0.77 (3H, t, *J* = 7.5 Hz, H-23), 1.12/1/34 (2H, m, H-4)] in **8** was substituted for a vinyl group [δ_H_ 5.60 (dd, *J* = 17.4, 10.8 Hz, H-4), 4.90 (dd, *J* = 10.8, 1.1 Hz, H-23a), 4.88 (dd, *J* = 17.4, 1.1 Hz, H-23)] in **5**. The ^1^H- and ^13^C-NMR (Table 2), HMBC (Figure 2), COSY (Figure 2), and NOESY (Figure 2) were compatible with the structure of **8** as 2-hydroxyl-3,4-seco-friedelan-3-oic acid ethyl-ester (**8**) [23].

In summary, *Actinobacteria* have been accepted as a big microbial bank that can be expected to provide a wide variety of structurally unique and biologically potent natural metabolites. In continuation of our previous chemical and biological investigations of microorganism-generated metabolites, a new *Actinobacteria* strain, identified as *Herbidospora daliensis*, isolated from a sediment soil sample, was ascertained to be able to produce bioactive metabolites during its solid fermentation according to our systematic screening program. The secondary metabolites of *Herbidospora* genus have rarely been studied in the past. The strain *H*. *daliensis* in this study has only been reported by our team for one component in the past [15]. After modification of the fermentation conditions, we obtained 8 components from the ethyl acetate active layer, 5 of which were new compounds, and the skeleton of the compounds covered benzofuran, coumarin, biphenyl and 3,4-seco-friedelane metabolites. These components were first discovered from the genus *Herbidospora*, which has chemical taxonomic significance. These results suggest that *Herbidospora* has distinct and diverse metabolites that arise under different fermentation conditions and soil-derived collections. It may therefore be possible to find more new bioactive natural products by searching the *Herbidospora* species under a special eco-environment.

### 2.2. Biological Studies

The 8 isolates in sufficient amounts were evaluated by examining their inhibitory effects on LPS-induced inducible nitric oxide synthase (iNOS)-dependent NO production in the murine macrophage cell line RAW 264.7 (Table 3). The inhibitory activity data of the 8 isolated compounds on NO generation by macrophages are shown in Table 3. Compared to quercetin (*IC*_50_ value 36.8 ± 1.3 μM), which was used as the positive control in this study, herbidosporadalins A, B, G & H (**1**, **2**, **6**, & **8**) exhibited NO inhibitory activity with *IC*_50_ values of 11.8 ± 0.9, 7.1 ± 2.9, 17.8 ± 1.7 & 13.3 ± 6.5, respectively. Compounds **1**, **2**, **8**, & **6** showed about 3, 5, 2 and 3-fold NO inhibitory activities compared to quercetin, respectively. Compounds **3** and **7** showed weak NO inhibitory activity, whereas compounds **4** and **5** displayed no NO inhibitory activities.

From the results of our above tests, the following conclusions can be drawn. Compound **6** (3,4-seco-friedelan analogs, herbidosporadalin F), with an ethyl 3-propanoate at C-10, exhibited more effective inhibition than its analogue, compound **8** (2-hydroxyl-3,4-seco-friedelan-3-oic acid ethyl-ester), with an ethyl-2-hydroxypropanoate group, and compound **5** (herbidosporadalin E), with a vinyl groups at C-5 against LPS-induced NO generation. Compound **5** (herbidosporadalin E), with a double bond between C-4 &23 substituent, exhibited less effective inhibition than its analogue, compounds **6** & **8**. The presence of ethyl groups on the C-4 position of the 3,4-seco-friedelans seem to play an important role in anti-inflammatory activity. Furthermore, the RT-PCR analysis in the present study indicated that LPS treatment increased the level of iNOS mRNA expression, and that compounds (**1**, **2**, **6**, & **8**) inhibited this increase in a concentration-dependent manner. At the highest concentration, none of the compounds tested showed any obvious cytotoxicity toward RAW 264.7 cells. Cytotoxic effects were measured using MTT assay. The high cell viability (>95%) indicated that the inhibitory activities of LPS-induced NO production by active compounds (**1**, **2**, **6**, & **8**) did not result from its cytotoxicity.

## 3. Materials and Methods

### 3.1. General Experimental Procedures

Column chromatography (CC): silica gel 60 (70–230 or 230–400 mesh, Merck, Meguro City, Tokyo, Japan) and Reversed Phase Silica Gel (RP-18) (particle size: 20–40 μm) (Silicycle, Québec, QC, Canada). TLC: silica gel 60 F_254_ precoated plates (Merck) and Spherical C18 100A IR Spectra were measured on a Perkin-Elmer-2000 FT-IR spectrophotometer; ^1^H-, ^13^C- and 2D-NMR spectra were run on Varian-Mercury-500 using chloroform-d as the solvent; EI-MS: VG-Biotech Quatro-5022 mass spectrometer; *m*/*z* (rel. %). HR-EI-MS spectra were recorded on a Finnigan/Thermo Quest NAT mass spectrometer. UV spectra were run on a Jasco UV-240 spectrophotometer, λ_max_ (log ε) in nm. Optical rotation: Jasco DIP-370 polarimeter; in CHCl_3_. HPLC: spherical C18 column (250 × 10 mm, 5 μm) (Waters, Milford, MA, USA) and LDC-Analytical-III apparatus.

### 3.2. Microorganism, Cultivation, and Preparation of the Actinobacteria Strain

The *Actinobacteria*, *Herbidospora daliensis* (0385M-1^T^), was isolated from sediment collected from the Dali area of Taiwan using HVY agar and was then cultured at 45 °C during 7 days. This *Actinobacteria* was identified by Mrs. Min Tseng, and the specimens (0385M-1^T^) were deposited at the Bioresource Collection and Research Center, Food Industry Research and Development Institute. Strains are maintained on oat agar and spore or mycelial suspensions are harvested with 20% (*v*/*v*) glycerol and stored at −20 °C. Mature slant cultures of strain 0385M-1T were inoculated into 500 mL flasks containing 100 mL of seed medium composed of 0.4% glucose, 0.4% yeast extract, and 1% malt extract (pH 7.0). After 4 days of growth at 30 °C on a rotary shaker (200 rpm), an aliquot (2 mL) of the seed culture was tranferred to 500 mL of production medium (Humic acid 1.0 g, KCl 1.7 g, FeSO_4_·7H_2_O 0.01 g, Na_2_HPO_4_ 0.5 g, CaCO_3_ 0.04 g, MgSO_4_·7H_2_O 0.05 g, yeast extract 1.1 g, Agar 20.0 g, dist. water 1.0 L, pH 7.4). After 21 days of cultivation at 30 °C temperature on a rotary shaker (200 rpm), the culture filtrates were obtained by filtering through filter paper.

### 3.3. Isolation and Characterization of Secondary Metabolites

Fermented broth (10 L) was filtered to separate the mycelium and culture broth. The culture broth was repeatedly extracted five times with EtOAc. The EtOAc layers were combined and dried to give EtOAc-soluble fraction (47.3 g). The EtOAc fraction (17.3 g) was applied to a silica gel column (230–400 mesh, 800 g), eluting with a gradient of *n*-hexane/EtOAc to give 6 fractions (1-6). Fraction 1 (1.1 g) was applied to a silica gel (230–400 mesh, 35 g), eluting with a gradient of *n*-hexane/acetone to give five fractions (1-1–1-5). Fraction 1-3 (211 mg) was chromatographed on a CC (6 g, SiO_2_, 230–400 mesh; *n*-hexane/EtOAc 40:1) to afford **1** (3.6 mg) and **3** (4.9 mg). Fraction 1-5 (70 mg) was applied to CC (1.5 g, SiO_2_, 230–400 mesh; n-hexane/Me_2_CO 5:1) to afford **2** (5.8 mg). Fraction 3 (8.25 g) was chromatographed on a silica gel column (230–400 mesh, 240 g), eluting with a gradient of *n*-hexane/acetone, to give 12 fractions (3-1–3-12). Fr. 3-5 (188.6 mg) was chromatographed on an RP-18 column (6 g), eluting with (acetone/H_2_O, 2.5:1) to obtain six fractions (Fr. 3-5-1–3-5-6). Fr. 3-5-2 (321.2 mg) was applied to a preparative RP-18 TLC (MeOH/H_2_O, 10:1) to afford **8** (0.8 mg). Fr. 3-7 (168 mg) was applied to silica gel, eluting with CH_2_Cl_2_/acetone (50:1) to afford **5** (2.8 mg). Fr. 3-8 (234.1 mg) was applied to a silica gel column (230-400 mesh, 10 g), eluting with *n*-hexane/EtOAc (2:1) to give six fractions (3-8-1–3-8-6). Fr. 3-8-3 (43.6 mg) was applied to a silica gel column (230–400 mesh, 1.5 g), eluting with CH_2_Cl_2_/acetone (50:1) and purified further by preparative TLC (CH_2_Cl_2_-acetone, 20:1) to obtain **4** (2.8 mg). Fr. 3-9 (216 mg) was applied to a silica gel column (230–400 mesh, 6 g), eluting with (CH_2_Cl_2_-acetone, 20:1) to give eight fractions (3-9-1–3-9-8). Fr. 3-9-5 (15.8 mg) was subjected to preparative HPLC (acetonitrile/H_2_O, 5:1) to afford **6** (2.5 mg) and **7** (4.8 mg).

Herbidosporadalin A (**1**): amber oil; [α${]}_{D}^{25}$ = +111.82 (*c* 0.01, CHCl_3_); IR (Neat): 3482 (OH), 1725 (ester), 1593, 1407 (aromatic ring) cm^−1^; ^1^H NMR (500 MHz, CDCl_3_): see Table 1; ^13^C NMR (125 MHz, CDCl_3_): see Table 2; EIMS (70 eV) *m*/*z* (%): 318 ([M]^+^, 7), 274 (11), 220 (31), 202 (100),174 (12); HREIMS *m*/*z* 318.1105 [M]^+^ (calcd. for C_17_H_18_O_6_, 318.1103).

Herbidosporadalin B (**2**): yellowish oil; [α${]}_{D}^{25}$ = 0 (*c* 0.01, CHCl_3_); UV (MeOH): 321 (4.45), 258 (3.94), 248 (3.91), 222 (4.30) nm; IR (Neat): 3516 (OH), 1732 (CHO), 1606, 1566, 1500 (aromatic ring) cm^−1^; ^1^H NMR (500 MHz, CDCl_3_): see Table 1; ^13^C NMR (125 MHz, CDCl_3_): see Table 2); EIMS (70 eV) *m*/*z* (%): 276 ([M]^+^, 100), 205 (4) ([M-CCHO(CH3)2]^+^, 175 (23); HREIMS *m*/*z* 276.0995 [M]^+^ (calcd. for C_1__5_H_16_O_5_, 276.0997).

Herbidosporadalin C (**3**): oil; UV (MeOH): 287 (4.39), 265 (4.60), 212 (4.59) nm; IR (Neat): 3390 (OH), 1598, 1504 (aromatic ring) cm^−1^; ^1^H NMR (500 MHz, CDCl_3_): see Table 1; ^13^C NMR (125 MHz, CDCl_3_): see Table 2); EIMS (70 eV) *m*/*z* (%): 332 ([M]^+^, 27), 317 (100), 306 (23), 158 (12); HREIMS *m*/*z* 332.1429 [M]^+^ (calcd. for C_22_H_20_O_3_, 332.1412).

Herbidosporadalin D (**4**): amorphous powder; mp. 177–179 °C; UV (MeOH): 310 (4.30), 281 (4.3), 230 (4.5) nm; IR (Neat): 3353 (OH), 1593 (CO) cm^−1^; ^1^H NMR (500 MHz, CDCl_3_): see Table 1; ^13^C NMR (125 MHz, CDCl_3_): see Table 2; EIMS (70 eV) *m*/*z* (%): 304([M]^+^, 50), 287 (33), 181 (100), 123 (13); HREIMS *m*/*z* 304.0937 [M]^+^ (calcd. for C_16_H_16_O_6_, 304.0947).

Herbidosporadalin E (**5**): amorphous solid; mp. 197–198 °C; [α${]}_{D}^{25}$ = +19.5 (*c* 0.01, CHCl_3_); IR (KBr): 1737 (OCO) cm^−1^; ^1^H NMR (500 MHz, CDCl_3_): see Table 1; ^13^C NMR (125 MHz, CDCl_3_): see Table 2; EIMS (70 eV) *m*/*z* (%): 470 ([M]^+^, 14), 1273 (15), 205 (100), 95 (73); HREIMS *m*/*z* 470.4117 [M]^+^ (calcd. for C_32_H_54_O_2_, 470.4124).

Herbidosporadalin F (**6**): white needles; mp. 185–186 °C; [α${]}_{D}^{25}$ = +5.76 (*c* 0.01, CHCl_3_); IR (Neat): 1737 (OCO) cm^−1^; ^1^H NMR (500 MHz, CDCl_3_): see Table 1; ^13^C NMR (125 MHz, CDCl_3_): see Table 2; EIMS (70 eV) *m*/*z* (%): 131 ([M + H]^+^, 41), 72 (100); HREIMS *m*/*z* 472.4276 [M + H]^+^ (calcd. for C_32_H_56_O_2_, 472.4280).

5-Hydroxy-6-[(2′*Z*)-4′-acetoxy-3′-methylbut-2′-enyl]-7-methoxy-2-methylchromone (**7**): oil; UV (MeOH): 291 (3.97), 256 (4.27), 251 (sh) (4.27), 232 (4.33) nm; IR (Neat): 3416 (OH), 1660 (conjugated ketone group), 1626, 1493 (benzene) cm^−1^; ^1^H NMR (500 MHz, CDCl_3_): see Table 1; ^13^C NMR (125 MHz, CDCl_3_): see Table 2; EIMS (70 eV) *m*/*z* (%): 290 ([M]^+^, 2), 272 (35), 257 (58), 231(52), 189 (54); HREIMS *m*/*z* 290.1144 [M]^+^ (calcd. for C_16_H_18_O_5_, 290.1154).

2-Hydroxyl-3,4-seco-friedelan-3-oic acid ethyl-ester (**8**): amorphous solid; mp. 211–212 °C; [α${]}_{D}^{25}$ = −0.32 (*c* 0.01, CHCl_3_); IR (KBr): 3432 (OH), 1752 (OCO) cm^−1^; ^1^H NMR (500 MHz, CDCl_3_): see Table 1; ^13^C NMR (125 MHz, CDCl_3_): see Table 2; EIMS (70 eV) *m*/*z* (%): 234 ([M]^+^, 19), 149 (100), 121 (13); HREIMS *m*/*z* 488.4227 [M]^+^ (calcd. for C_32_H_56_O_3_, 488.4229).

### 3.4. Determination of NO Production and Cell Viability Assay

Mouse macrophage cell line (RAW 264.7) was obtained from the Bioresource Collection and Research Center (BCRC 60001) and cultured at 37 °C in Dulbecco’s Modified Eagle’s Medium (DMEM) supplemented with 10% fetal bovine serum (Gibco, Shanghai, China), 4.5 g/L glucose, 4 mM glutamine, penicillin (100 units/mL), and streptomycin (100 μg/mL) in a humidified atmosphere in a 5% CO_2_ incubator. The cells were treated with 10, 25, 50 μM natural products in the presence of 1 μg/mL LPS (lipopolysaccharide, Sigma-Aldrich, St. Louis, MO, USA) for 20 h. The concentration of NO in culture supernatants was determined as nitrite, a major stable product of NO, by Griess reagent assay [24], and cell viabilities were determined using the MTT assay as described previously [25].

## 4. Conclusions

*Actinobacteria* have great economic and biotechnological value and have long been recognized as the main microorganisms in the medical industry. To date, there are tens of thousands of antibiotics produced by microorganisms, of which more than 70% are derived from *Actinobacteria* [26]. Secondary metabolites of *Actinobacteria* have various structures and biological activities, including antibacterial, antifungal, antitumor, insecticidal and herbicidal, enzyme inhibition, immune regulation, etc. [27], which indicates that *Actinobacteria* have great potential for the development of new medicines. As part of our investigations aimed at exploring structurally novel bioactive secondary metabolites from actinomycetes, chemical research on the fermentation extract of the *H*. *daliensis* led to the isolation of seven previously undescribed compounds, namely, herbidosporadalins A–G (**1**–**7**), and one compound isolated from nature for the first time, namely, herbidosporadalin H (**8**) (Figure 1). The structure of these isolates was determined by spectroscopic experiments. The EtOAc soluble fraction from the *H*. *daliensis* fermentation broth was tested in vitro and showed anti-inflammatory activity that decreased the LPS-stimulated nitric oxide (NO) in RAW 264.7 cells. In addition, compounds **1**, **2**, **7**, and **8** showed potent inhibition with IC_50_ values ≤ 17.8 μM, against lipopolysaccharide (LPS)-induced nitric oxide (NO) generation, stronger than the positive control quercetin (IC_50_ = 36.8 ± 1.3 μM). To the best of our knowledge, this is the first report on 3,4-seco-friedelane metabolites (**6**–**8**) from the genus *Herbidospora*.

In previous surveys, there have been many reports on the activity of actinomycete metabolites in the literature, but few reports of active natural products were evaluated. The current study reported the inhibitory activities of active natural products against nitric oxide production. Therefore, it is worth continuing to study the effect of such substances upon other various pro-inflammatory cytokines, such as interleukin (IL)-12p40, IL-6, and tumor necrosis factor (TNF)-α.

## Figures and Tables

**Table 1:** ^1^H NMR data for Compounds **1**–**8** in CDCl_3_ (*δ* in ppm, *J* in Hz, 500 MHz in CDCl_3_).

No	1	2	3	4	5	6	7	8
1			7.40 (d, *J =* 2.0)		1.38/1.53 (each 1H, m)	1.48/1.53 (each 1H, m)		1.65/1.48 (each 1H, m)
2					2.25 (m)	2.29 (t, *J* = 8.7)		4.07 (m)
Me-2							2.34 (s)	
3	6.35 (d, *J =* 9.6)	6.25 (d, *J =* 9.6)	7.06 (t, *J =* 2.0)	6.08 (s)			6.03 (s)	
4	7.75 (d, *J =* 9.6)	7.62 (d, *J =* 9.6)			5.60 (dd *J =* 17.4, 10.8)	1.06/1.32 (each 1H, m)		1.12/1.34 (each 1H, m)
5	7.36 (s)	7.41 (d, *J =* 8.8)		6.08 (s)				
OH-5							13.05 (s)	
6		6.87 (d, *J =* 8.8)			1.37/1.44 (each 1H, m)	1.46/1.13 (each 1H, m)		1.48/1.18 (each 1H, m)
Me-6			1.65 (s)					
7			7.13 (d, *J =* 7.9)		1.38/1.43 (each 1H, m)	1.34/1.38 (each 1H, m)		1.37/1.40 (each 1H, m)
OMe-7							3.89 (s)	
8			7.12 (d, *J =* 7.9)		1.30 (m)	1.21 (m)	6.38 (s)	1.27 (m)
9								
Me-9			2.38 (s)					
10			7.57 (s)		0.87 (m)	0.79 (m)		1.33 (m)
11					1.41/1.38 (each 1H, m)	1.39/1.36 (each 1H, m)		1.39/1.36 (each 1H, m)
12					1.28/1.33 (each 1H, m)	1.27/1.30 (each 1H, m)		1.37/1.35 (each 1H, m)
15					1.46/1.26 (each 1H, m)	1.45/1.25 (each 1H, m)		1.46/1.27 (each 1H, m)
16					1.54/1.35 (each 1H, m)	1.51/1.32 (each 1H, m)		1.52/1.32 (each 1H, m)
18						1.51 (m)		1.51 (m)
19					1.34/1.17 (each 1H, m)	1.33/1.17 (each 1H, m)		1.34/1.17 (each 1H, m)
21					1.45/1.25 (each 1H, m)	1.42/1.23 (each 1H, m)		1.45/1.26 (each 1H, m)
22					1.45/0.91 (each 1H, m)	1.45/0.91 (each 1H, m)		1.45/0.91 (each 1H, m)
23					4.90 (dd, *J* = 10.8, 1.1), 4.88 (dd, *J* = 17.4, 1.1)	0.75 (t, *J* = 7.4)		0.77 (t, *J* = 7.5),
24					0.96 (s)	0.75 (s)		0.80 (s)
25					0.86 (s)	0.84 (s)		0.79 (s)
26					0.97 (s)	0.95 (s)		0.95 (s)
27					0.99 (s)	0.97 (s)		1.00 (s)
28					1.15 (s)	1.14 (s)		1.14 (s)
29					0.92 (s)	0.91 (s)		0.92 (s)
30					0.97 (s)	0.96 (s)		0.97 (s)
31					4.08 (q, *J* = 7.2)	4.08 (q, *J* = 7.1)		4.08 (m)
32					1.23 (t, *J* = 7.2)	1.23 (t, *J* = 7.1)		1.23 (t, *J* = 7.1)
1′		5.52 (s)					3.40 (d, *J =* 8.0)	
2′	7.68 (d, *J =* 2.0)		7.48 (d, *J =* 8.5)	7.59 (d, *J =* 1.6)			5.32 (t, *J =* 8.0)	
CHO-2′		9.71 (s)						
3′	6.80 (d, *J =* 2.0)	1.06 (s)	6.88 (dt, *J =* 8.5)					
Me-3′							1.77 (s)	
4′		1.17 (s)					4.25 (s)	
5′			6.88 (d, *J =* 8.5)	6.84 (d, *J =* 8.3)				
6′			6.88 (d, *J =* 8.5)	7.22 (dd, *J =* 8.3, 1.6)				
1″	4.37 (dd, *J* = 10.0, 8.4) 4.71 (dd, *J* = 10.0, 2.8)							
2″	4.00 (dd, *J* = 8.4, 2.8)							
4″	1.26 (s)							
5″	1.26 (s)							
OMe-2″	3.24 (s)							
OMe-2,6				3.63 (s)				
OMe-3′				3.93 (s)				

**Table 2:** ^13^C NMR data for Compounds **1**–**8** (*δ* in ppm, 125 MHz for ^13^C NMR in CDCl_3_).

No	1	2	3	4	5	6	7	8
1			112.4	110.4	21.3	21.0		38.8
2	160.0	159.3	134.0	158.6	37.4	37.5	165.9	71.8
3	114.7	113.3	112.9	92.0	173.8	173.8	108.6	175.9
4	144.3	143.2	145.8	158.5	151.0	35.9	181.7	36.1
4a	116.5	113.0					105.6	
5	113.5	128.4		92.0	42.0	37.8	157.4	38.0
6	126.0	107.8	78.8	150.5	41.5	38.9	111.4	38.9
7	148.2	159.3	123.2	194.2	17.9	18.1	162.1	18.1
OMe-7		56.2					56.1	
8	131.9	114.8	128.9		53.0	53.0	89.7	52.8
8a	143.5	151.9					156.0	
9			137.4		38.6	39.0		38.2
10			123.1		58.3	59.8		54.3
11			128.2		35.1	35.1		35.1
12			136.4		30.2	30.2		30.2
13			122.6		39.6	39.6		39.5
14			138.5		38.3	38.3		38.5
15					32.2	32.2		32.3
16					36.0	36.0		36.1
17					29.9	29.9		30.1
18					42.7	42.7		42.8
19					35.2	35.3		35.3
20					28.1	28.1		28.1
21					32.7	32.8		32.8
22					39.2	39.2		39.2
23					110.7	7.6		7.6
24					18.1	19.3		19.5
25					18.0	17.9		18.4
26					18.7	20.1		18.7
27					20.1	18.7		20.1
28					32.0	32.1		32.1
29					34.9	34.9		35.0
30					31.8	31.8		31.8
31					60.0	60.1		61.4
32					14.2	14.2		14.2
1′		72.0	134.5	131.1			21.4	
2′	146.8	52.8	128.1	110.1			124.7	
3′	106.8	18.4	115.5	146.5			134.8	
4′		20.0	154.8	150.5			61.7	
5′			115.5	113.6				
6′			128.1	126.2				
1″	75.6							
2″	76.0							
3″	75.5							
4″	21.4							
5″	20.6							
OMe-2″	49.3							
CHO		203.8						
Me-3′							22.5	
Me-6			27.7					
Me-9			21.2					
OMe-2,6				55.7				
OMe-3′				56.0				

**Table 3:** Inhibitory effects of the isolates (**1**–**8**) on NO generation by RAW 264.7 murine macrophages in response to lipopolysaccharide (LPS).

Compounds	IC_50_ (μM) ^(a)^
	NO
herbidosporadalin A (**1**)	11.8 ± 0.9
herbidosporadalin B (**2**)	7.1 ± 2.9
herbidosporadalin C (**3**)	75.5 ± 11.5
herbidosporadalin D (**4**)	>100
herbidosporadalin E (**5**)	>100
herbidosporadalin F (**6**)	13.3 ± 6.5
5-hydroxy-6-[(2′Z)-4′-acetoxy-3′-methylbut-2′-enyl]-7-methoxy-2-methylchromone (7)	65.5 ± 4.8
2-hydroxyl-3,4-seco-friedelan-3-oic acid ethyl-ester (8)	17.8 ± 1.7
Quercetin ^(b)^	36.8 ± 1.3

^(a)^ The IC_50_ values were calculated from the slope of the dose–response curves (*SigmaPlot*). Values are expressed as mean ± S.E.M. of three independent experiments. ^(b)^ Quercetin was used as a positive control.

## Data Availability

Not applicable.

## Sample Availability

Samples of the compounds **1**–**4** are available from the authors.
